# Biomechanical Effects of Posterior Condylar Offset and Posterior Tibial Slope on Quadriceps Force and Joint Contact Forces in Posterior-Stabilized Total Knee Arthroplasty

**DOI:** 10.1155/2017/4908639

**Published:** 2017-11-19

**Authors:** Kyoung-Tak Kang, Yong-Gon Koh, Juhyun Son, Oh-Ryong Kwon, Jun-Sang Lee, Sae Kwang Kwon

**Affiliations:** ^1^Department of Mechanical Engineering, Yonsei University, 50 Yonsei-ro, Seodaemun-gu, Seoul 03722, Republic of Korea; ^2^Joint Reconstruction Center, Department of Orthopaedic Surgery, Yonsei Sarang Hospital, 10 Hyoryeong-ro, Seocho-gu, Seoul 06698, Republic of Korea

## Abstract

This study aimed to determine the biomechanical effect of the posterior condylar offset (PCO) and posterior tibial slope (PTS) in posterior-stabilized (PS) fixed-bearing total knee arthroplasty (TKA). We developed ±1, ±2, and ±3 mm PCO models in the posterior direction and −3°, 0°, 3°, and 6° PTS models using a previously validated FE model. The influence of changes in the PCO and PTS on the biomechanical effects under deep-knee-bend loading was investigated. The contact stress on the PE insert increased by 14% and decreased by 7% on average as the PCO increased and decreased, respectively, compared to the neutral position. In addition, the contact stress on post in PE insert increased by 18% on average as PTS increased from −3° to 6°. However, the contact stress on the patellar button decreased by 11% on average as PTS increased from −3° to 6° in all different PCO cases. The quadriceps force decreased by 14% as PTS increased from −3° to 6° in all PCO models. The same trend was found in patellar tendon force. Changes in PCO had adverse biomechanical effects whereas PTS increase had positive biomechanical effects. However, excessive PTS should be avoided to prevent knee instability and subsequent failure.

## 1. Introduction

Total knee arthroplasty (TKA) is one of the most successful orthopedic surgical treatments for providing pain relief and improving knee function. It has reported survival rates exceeding 90% after 15 years [1, 2]. The fundamental goal of TKA is to reduce knee joint pain and maintain the range of motion (ROM) to facilitate the ability to perform daily activities [3]. With remarkable improvements in implant design and survival, most patients primarily consider objective functional outcomes such as knee kinematics to evaluate the success of TKA [4]. A minimum ROM of 90° is required for daily activities; higher-level activities, such as running and cycling, depend on increased ROM [5]. A recent study demonstrated that limited ROM is negatively correlated with patient satisfaction and functional ability after TKA [6].

During the past decade, many studies dealt with femoral posterior condylar offset (PCO) and posterior tibial slope (PTS) in TKA [7–12]. Various studies demonstrated the importance of PCO for attaining ROM in cruciate-retaining (CR) TKA [7–10]. However, while PCO is considered irrelevant to the knee ROM in posterior-stabilized (PS) TKA, the influence of other factors on knee ROM has not been considered [8, 9, 11, 12]. Previous studies on this subject had some limitations. All of the factors that are present before (e.g., the physical conditions of the patients), during (e.g., surgical techniques and implant design), and after (e.g., complications and rehabilitation procedures) TKA can affect the postoperative results [13–15]. However, most studies did not consider the impact of these factors when analyzing the correlation between PCO and ROM. In addition, the difference in the weight-bearing status can affect the flexion angle [15–17]. Even under the same weight-bearing status, active and passive ROM may also differ significantly [17, 18]. Shi et al. reported that an increase in the PTS can significantly increase the postoperative maximal knee flexion in PS TKA [19]. Okamoto et al. reported that the maximum quadriceps force and patellofemoral (PF) contact force decreased with increasing PTS [20]. They supported the suggestion that increased PTS contributes to improved exercise efficiency during knee extension; however, excessive PTS should be avoided to prevent knee instability [20]. PCO and PTS lead to diverse biomechanical effects, as mentioned above, and there still exists no consensus as the results depend on the CR and PS types of TKA.

The advantage of computational simulation using a single subject is that we can determine the effects of component alignment within the same subject without any effect of variables such as weight, height, bone geometry, ligament properties, and component size [21]. Moreover, to the best of our knowledge, no study has evaluated the biomechanical effects with respect to changes in PCO and PTS by considering the contact stress in the polyethylene (PE) insert and patellar button and the forces on the quadriceps muscle and patellar tendon.

The purpose of this study was to evaluate the biomechanical effects with respect to changes in PCO and PTS in PS TKA. We analyzed the contact stress in the PE insert and patellar button and the forces on the quadriceps muscle and patellar tendon by using a validated finite element (FE) model under the deep-knee-bend loading condition. We hypothesized that changes in PCO had smaller biomechanical effects, but changes in PTS lead to a positive effect.

## 2. Materials and Methods

The model used in this study is based on a previously validated and published knee joint FE model [22–25]. An FE model of the lower extremity was developed using the imaging data obtained from a healthy, skeletally mature young male athlete with no history of knee injury. The model includes the bony structures of the knee joint, including details of the soft tissues of the PF and tibiofemoral (TF) anatomy.

### 2.1. Intact Model Development

A three-dimensional (3D) nonlinear FE model of a normal knee joint was developed using data from the computed tomography (CT) and magnetic resonance imaging (MRI) scans of a healthy 37-year-old male subject. Scans were obtained while the subject was supine with the leg in an unloaded neutral position. The CT and MRI models were developed with a slice thickness of 0.1 mm and 0.4 mm, respectively.

The reconstructed MRI models were combined into CT model with the positional alignment by using commercial software (Rapidform version 2006, 3D Systems Korea, Inc., Seoul, Republic of Korea). The software models bone structures as rigid bodies using four-node shell elements [26]. The ligament insertion points were referenced to the anatomy from the MRI sets of the subject and descriptions found in literature [27–29]. In addition, the major ligaments were modeled with nonlinear, tension-only spring elements [30, 31].

### 2.2. Development of the Model for Changes in PCO and Increase in PTS

A total of 28 models were considered in this study. The surgical simulation of a TKA was performed by two experienced surgeons (the second and sixth authors). Computer-assisted design models of a PS design from the Genesis II Total Knee System (Smith & Nephew Inc., Memphis, TN, USA) were virtually implanted in the bone geometry. Based on the dimensions of the femur and tibia, devices of sizes 7 and 5-6 were selected for the femoral component and tibial baseplate, respectively. A reference “neutrally aligned model” was defined according to the conventional surgical guidelines by inserting the femoral and tibial components orthogonally to the mechanical axis. Based on the baseline model, the other 27 models with different planes for bone resections of the femur or the tibia or both were defined.

**Figure 1:**
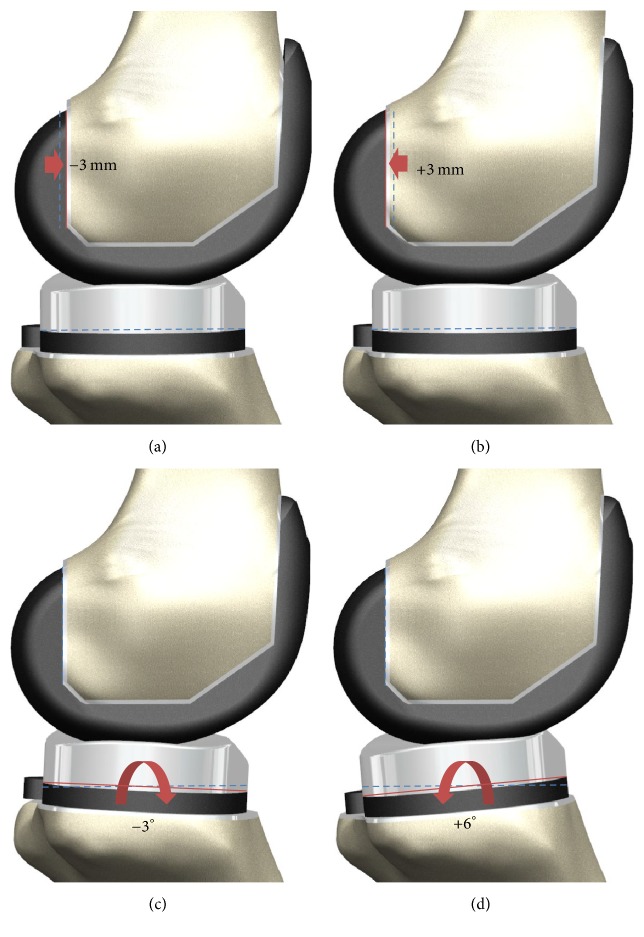
Schematic of the knee models used in this study: (a) PCO with −3 mm; (b) PCO with +3 mm; (c) PTS with −3°; (d) PTS with +6°.

In the neutral position, the femoral component was aligned such that the distal bone resection was perpendicular to the mechanical axis of the femur, and the anterior and posterior resections were parallel to the clinical epicondylar axis in the transverse plane. A PCO model identical to the original subject was developed by alignment of posterior condyles of the component and femur; subsequently, the modified PCO model was developed. Seven models were developed with −3, −2, −1, 0, +1, +2, and +3 mm in the posterior direction (Figure 1).

The tibial default alignment was rotated by 0° connecting the centers of the medial and lateral condyles, and the coronal alignment was 90° to the mechanical axis. The sagittal alignments were −3°, 0°, 3°, and 6° to the posterior slope, with an 8 mm resection below the highest point of the lateral plateau (Figure 1). This is the lowest point of the PE insert articular surface adjacent to the lowest points of the femoral articular surfaces in extension.

Contact conditions were applied between the femoral component, PE insert, and patellar button in TKA. The coefficient of friction between the PE material and metal was chosen as 0.04 for consistency with previous explicit FE models [32].

The materials of the femoral component, PE insert, tibial component, and bone cement corresponded to a cobalt chromium alloy (CoCr), ultrahigh-molecular-weight-polyethylene (UHMWPE), a titanium alloy (Ti6Al4V), and poly(methyl methacrylate) (PMMA), respectively. As in previous studies, these materials were assumed homogeneous and isotropic, except for the PE insert [32–36]. The material properties, in terms of Young's modulus (*E*) and Poisson's ratio (*v*), were as follows: CoCr:* E* = 220 GPa and* v* = 0.3; UHMWPE:* E* = 685 MPa and* v* = 0.47; Ti6Al4V:* E* = 110 GPa and* v* = 0.3; and PMMA:* E* = 1,940 MPa and* v* = 0.4 [32–36]. We considered a cement layer with a constant penetration depth of 3 mm into the bone according to the test for different cementing techniques at the femoral and tibial resection surfaces in contact with the femoral and tibial components, respectively [37, 38]. The interfaces between the prosthesis and bone were rigidly fixed considering the cement used [34, 39].

The FE models topologies provided 12 degrees of freedom in total to the TF and PF joints. The FE investigation included two types of loading conditions corresponding to the loads used in the experiments in the study for TKA model validation and model predictions under deep-knee-bend loading conditions. The intact model was validated in a previous study [22, 25], and the TKA model was validated by comparing it with the models used in a previous study [40]. A conservative ankle force of 50 N (reaction force) and hamstring forces of 10 N were constantly exerted with a linearly rising force and a maximum of approximately 600 N at 90° flexion of the quadriceps actuators for the TKA model under the first loading condition [40]. The second loading condition was deep-knee-bend (120°) loading, which was applied to evaluate the effect of the change in PCO and the corresponding PTS. The computational analysis was performed with an anterior-posterior force applied to the femur and a compressive load applied to the hip [25, 41, 42]. A proportional-integral-derivative controller was incorporated into the computational model to enable control of the quadriceps in a manner similar to that in a previous experiment [43]. The control system was used to calculate the instantaneous quadriceps displacement required to match a target flexion profile, which was the same as that in an experiment [43]. Through this, quadriceps force required for 120° flexion could be evaluated with respect to changes in PCO and PTS. Internal-external and varus-valgus torques were applied to the tibia [25, 41, 44].

The FE model was analyzed using the ABAQUS software (version 6.11; Simulia, Providence, RI, USA). The results for the average contact stress on the PE insert and patellar button were evaluated, and the forces on the patellar tendon and quadriceps were evaluated by analyzing the effect of changes in PCO and PTS.

### 2.3. Validation of the Intact and TKA Model

Our intact model was validated in previous study [22–24]. To validate the intact knee joint FE model, it was compared with the results from the experiment using subject from FE model. In the loading condition with 30° flexion, anterior tibial translation was measured to 2.83 mm in the experiment and 2.81 mm in the FE model; posterior tibial translation was measured to be 2.12 mm in the experiment and 2.09 mm in the FE model for validation. In experiments, the contact area was 246.8 mm^2^ and 188.5 mm^2^ in the medial and lateral articular cartilages, respectively. In FE model, the contact area was 255.7 mm^2^ and 199.2 mm^2^ in the medial and lateral articular cartilages, respectively. There was a good agreement between the experimental results and the FE model.

**Figure 2:**
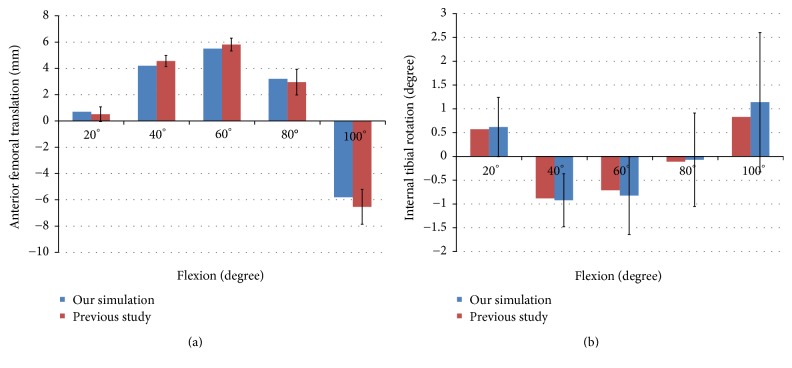
Comparison of (a) anterior femoral translation and (b) internal tibial rotation with previous experiments for validation of our simulation model. The error bars indicate 1 standard error.

TKA FE model was compared with previous experimental data for validation. The FE model for the femur was translated by 0.7 mm, 4.2 mm, 5.5 mm, 3.2 mm, and −5.8 mm in anterior direction at 20°, 40°, 60°, 80°, and 100° flexion, respectively (Figure 2(a)). In addition, The FE model for the tibia was rotated by 0.57°, −0.88°, −0.71°, −0.11°, and 0.83° in the internal rotation under 20°, 40°, 60°, 80°, and 100° flexion, respectively (Figure 2(b)). There was good agreement between the simulation results and those of a previous experimental study for the range of values under the loading condition applied to the prosthetic implant [40].

## 3. Results

### 3.1. Simulation of Contact Stresses on the PE Insert and Patellar Button with respect to Changes in PCO and Increase in PTS in the FE Models under Deep-Knee-Bend Conditions

**Figure 3:**
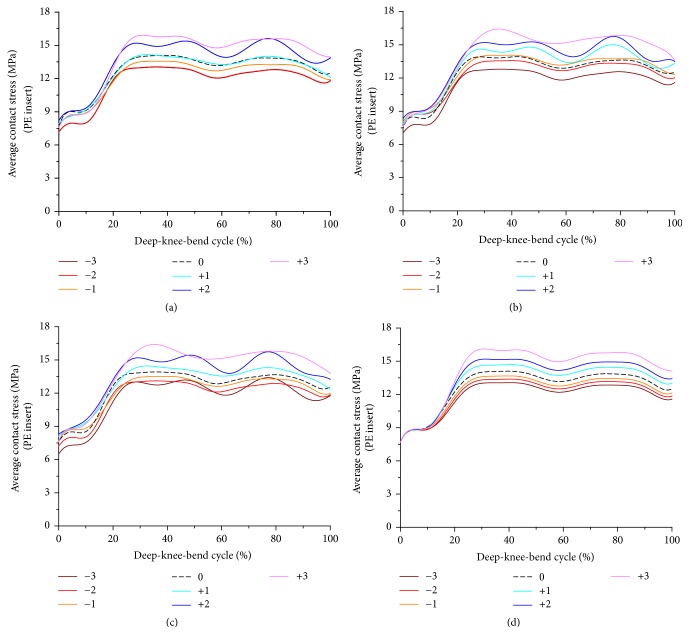
Comparison of average contact stress on the PE insert with respect to different PCO: (a) in the PTS −3°, (b) PTS 0°, (c) PTS +3°, and (d) PTS +6°.

Figure 3 showed the contact stress on the PE insert with respect to changes in PCO and increase in PTS. The contact stress on the PE insert increased and decreased as PCO increased and decreased, respectively, compared to the neutral position. This trend did not change regardless of the increase in PTS. The contact stress on the PE insert increased by 14% and decreased by 7% on average as the PCO increased by 3 mm and decreased by 3 mm, respectively, compared to the neutral position in all PTS cases. There was no difference in contact stress on the PE insert as PTS increased from the neutral PCO. This trend did not change regardless of the change of PCO in both anterior and posterior directions.

**Figure 4:**
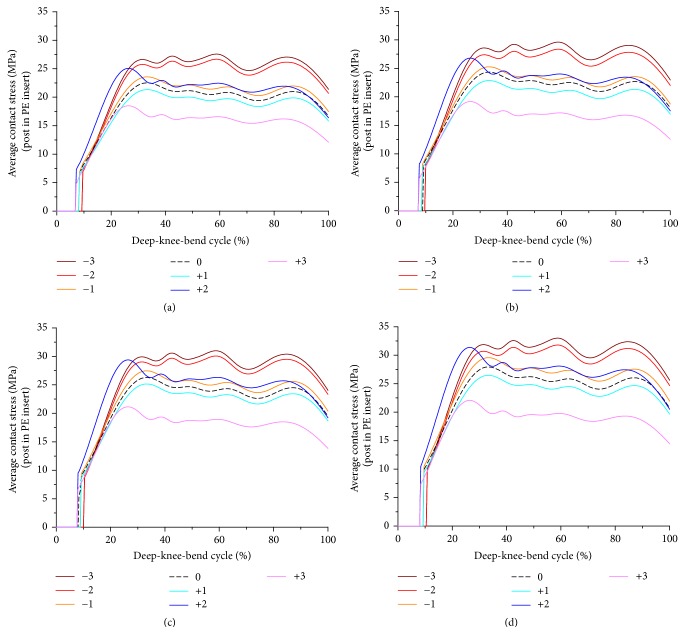
Comparison of average contact stress on the post in PE insert with respect to different PCO: (a) in the PTS −3°, (b) PTS 0°, (c) PTS +3°, and (d) PTS +6°.

Figure 4 showed the contact stress on the post in PE insert with respect to changes in PCO and increase in PTS. The contact stress on post in PE insert increased as PCO decreased and PTS increased. The contact stress on post in PE insert increased by 18% on average as PTS increased from −3° to 6° in all cases of PCO. It also increased by 26% on average, as compared to the neutral position, as PCO decreased by 3 mm in all cases of PTS cases.

**Figure 5:**
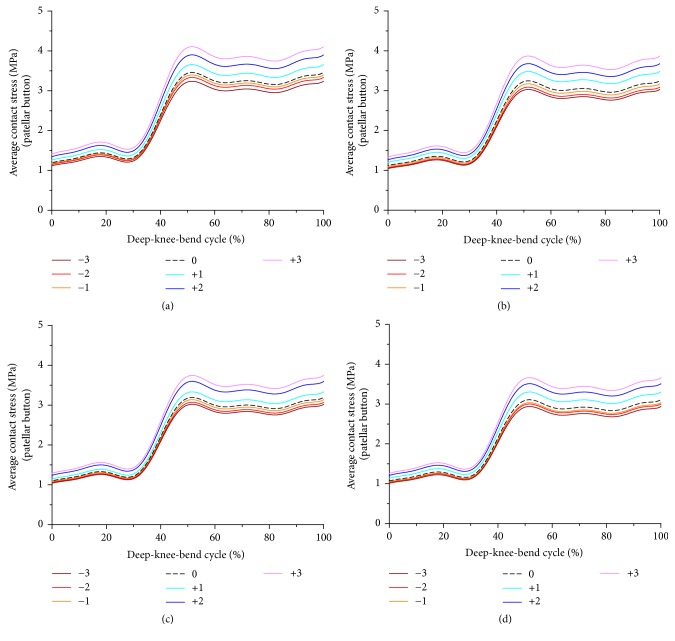
Comparison of average contact stress on the patellar button with respect to different PCO: (a) in the PTS −3°, (b) PTS 0°, (c) PTS +3°, and (d) PTS +6°.

Figure 5 showed the contact stress on the patellar button with respect to changes in PCO and increase in PTS. The contact stress on the patellar button showed the opposite trend for PCO, and it showed an identical trend for the PE insert based on PTS. The contact stress on the patellar button increased and decreased as PCO decreased and increased, respectively. In addition, it decreased as PTS increased in all PCO models. The contact stress on the patellar button increased by 8% and decreased by 6% on average as PCO increased by 3 mm and decreased by 3 mm, respectively, as compared to the neutral position in all PTS cases. The contact stress on the patellar button decreased by 11% on average as PTS increased from −3° to 6° in all different PCO cases.

### 3.2. Simulation of the Quadriceps Muscle Forces and Patellar Tendon with Change in PCO and Increased PTS in FE Models for Deep-Knee-Bend Conditions

**Figure 6:**
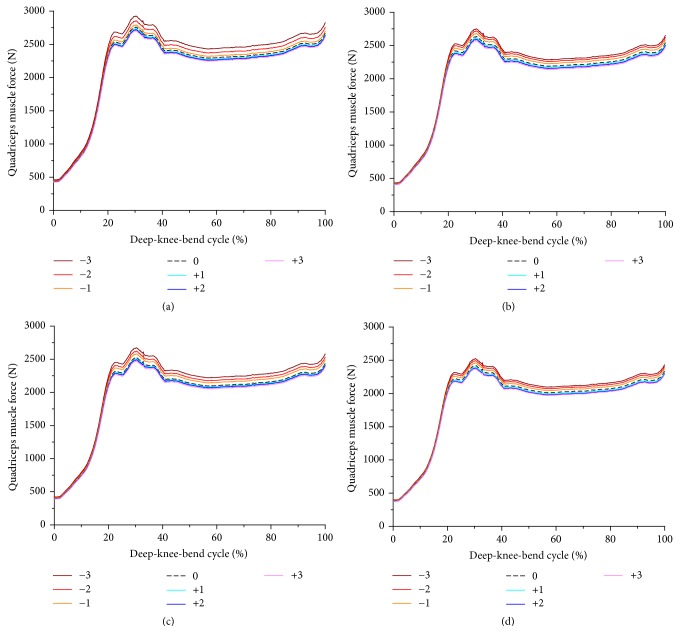
Comparison of the quadriceps muscle force with respect to different PCO: (a) in the PTS −3°, (b) PTS 0°, (c) PTS +3°, and (d) PTS +6°.

**Figure 7:**
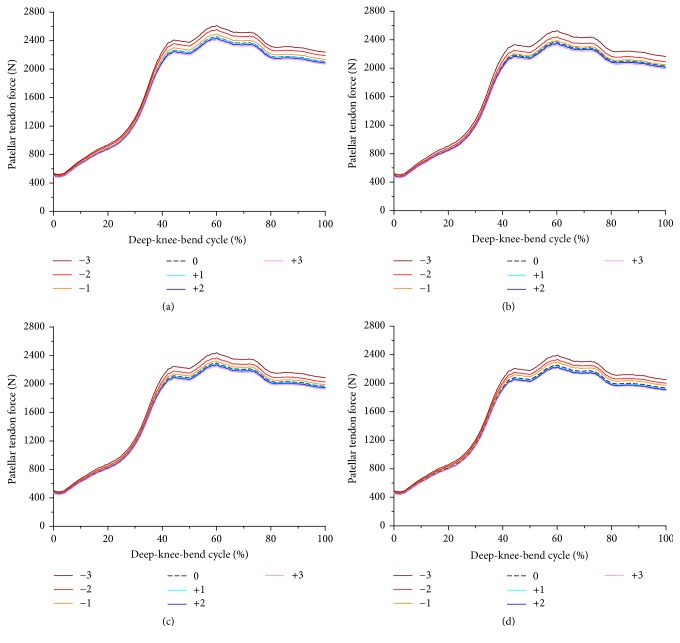
Comparison of the patellar tendon force with respect to different PCO: (a) in the PTS −3°, (b) PTS 0°, (c) PTS +3°, and (d) PTS +6°.

The quadriceps muscle forces and patellar tendon forces with changes in PCO and an increase in PTS under the deep-knee-bend condition are shown in Figures 6 and 7. The PCO changes to the anterior direction and decrease in PTS necessitated the highest force of the lumped quadriceps, up to 120° of knee flexion, under the deep-knee-bend conditions. The average quadriceps muscle forces increased by 5.3% and decreased by 2.1% upon translation in −3 anterior and +3 posterior directions, respectively, compared to the neutral position in all PTS models. The average force on the patellar tendon increased by 6.3% and decreased by 1.8% upon translation in −3 anterior and +3 posterior directions, respectively, as compared to the neutral position in all PTS models. In addition, the forces of the quadriceps muscle and patellar tendon decreased by 14% and 11% on average, respectively, as PTS increased from −3 to +6 in all PCO models.

## 4. Discussion

The most important finding of this study was that different biomechanical effects were observed with respect to the change of PCO in the anterior and posterior directions and that increased PTS led to a positive biomechanical effect in the knee joint, because the quadriceps force and patellar tendon force that were required to lead 120° flexion decreased as PTS increased. However, the excessive increase in PTS should be avoided to prevent failure in post on PE insert. In addition, the biomechanical effect due to changes in PCO was smaller than that due to the increased PTS because of the post-cam contact mechanism in PS TKA.

Since Bellemans et al. [7] proposed the impingement in the posterior region of the knee joint, it has gradually been accepted that PCO is important for better ROM in CR TKA [7–10, 42]. The potential correlation of PCO with ROM in PS TKA, however, remains debatable. Some studies reported that PCO could also affect the ROM in PS TKA [45, 46], whereas others suggested that such correlation was not statistically significant [8, 9, 11, 12]. Wang et al. suggested that such a difference could be caused by the different methods adopted for ROM measurement under weight-bearing and non-weight-bearing conditions [47]. Yang et al. hypothesized that full-thickness cartilage-based PCO is a valid criterion for comparing the preoperative and postoperative offsets, and it has a significant influence on postoperative ROM in TKA. Full-thickness cartilage-based PCO is an optimal criterion to estimate the changes in PCO before and after TKA [48]. However, in the study of Yang et al., neither cartilage-based nor radiographic PCO appeared to have a significant influence on the postoperative knee flexion after PS TKA. Soda et al. studied the relationship between the PCO ratio and postoperative flexion in PS TKA considering mainly a group of females [45]. They introduced a new parameter, namely, the posterior condylar offset ratio (PCOR), to eliminate any complication caused by the differences in the sizes of the distal femur among the patients [45]. Ishii et al. reported that the differences in individual PCO with current CR or PS TKA did not correlate with changes in knee flexion one year after TKA [49]. They recognized that correctly identifying the condyle that affects the results of the TKA may be difficult with the conventional radiographic techniques [49]. Recently, Antony et al. examined the influence of the sagittal plane component alignment to ROM [50].

As previously mentioned, the correlation between ROM and PCO in PS TKA is still controversial. In addition, most previous studies only considered PCO and ROM, but not PTS. PTS is also factor as important as PCO with regard to ROM [19]. However, to the best of our knowledge, no study has evaluated the forces on the quadriceps muscle and patellar tendon and the contact stress on the PE insert and patellar button for maximum ROM with respect to both PCO and PTS. Therefore, quadriceps force was evaluated during flexion to investigate the effect of change in PCO and PTS on ROM. In previous computational model, maximum flexion was assumed with implant-bone impingement [51]. However, muscle and ligament were not considered under flexion in previous study [51]. Combining its proven capabilities with the advantages of computer simulations was an effective means to perform our parametric investigation of the effects of change in PCO and PTS variability.

We investigated the biomechanical effect with respect to not only the changes in PCO but also an increase in PTS. We hypothesized that there was relatively less change in the biomechanical effect with respect to changes in PCO and that an increase in PTS had a positive biomechanical effect. To test this hypothesis, we evaluated the forces on the quadriceps muscle and patellar tendon and the contact stress on the PE insert and patellar button with respect to both PCO and PTS by using a previously validated computational knee joint model.

We found that the contact stress on the PE insert increased and decreased as PCO increased and decreased, respectively. The contact stress on the patellar button showed opposite trends in all PTS cases. The interesting finding was that as PCO decreased and increased, larger and smaller forces, respectively, for the quadriceps muscle corresponded to the same flexion angle. In addition, a similar trend was found in the case of the patellar tendon. The smaller force required for flexion as the PCO increased meant a greater advantage of the extensor mechanism, indicating a good agreement with the results of previous studies [45, 46]. However, PCO had a smaller influence than PTS.

Previous 3D fluoroscopic analyses of TKA suggested that posterior femoral rollback with flexion was consistently exhibited in the case of PS TKA, while an anterior femoral translation with flexion was observed in the case of CR TKA [52]. Therefore, unlike the case for CR TKA, the post-cam mechanism in PS TKA can theoretically prevent an anterior femoral translation during flexion, leading to posterior impingement even with decreased PCO [8]. In addition, our study showed that the forces on the quadriceps muscle required to provide identical flexion angles were less influenced by PCO decrease. It was found that the contact stress in post on PE insert increased as PCO decreased. Early engagement in post-cam occurred as femoral component was anteriorly located and PCO decreased leading to greater contact stress in post on PE insert. Increased PCO and PTS caused contact position between the tibiofemoral components posteriorly translated more, leading to an increase in the quadriceps lever arm, in turn improving the movement efficiency and contributing to reduced quadriceps force and PF contact stress.

Unlike the case for PCO, an increase in PTS had positive biomechanical effects. PTS provided consistent results regardless of the change in PCO. In a previous study, the differences in clinical outcomes between 0° and 5° PTS were compared, and it was reported that larger PTS did not influence the increasing postoperative ROM or improvement in the functional score with the Hospital for Special Surgery system [53]. However, it is understood that a greater PTS widens the flexion gap, which has already increased because of PCL removal, and if excessive PTS is allowed, the resulting posterior flexion instability can lead to a posterior substituting knee [54]. In addition, post-cam impingement occurs if the tibial component is placed with excessive PTS [55]. However, in this study, forces on the quadriceps muscles and patellar tendon required to cause identical maximum flexion decreased as PTS increased.

Catani et al. also found a significant correlation between PTS and the maximal flexion throughout in vivo video-fluoroscopic study on the knee joint kinematics in PS TKA [56]. Most clinical studies failed to find such a correlation between PTS and the maximal flexion [57, 58]. It seemed that many factors could affect the postoperative maximal flexion in the knee joint. However, it was difficult to provide objective evidence for the effect of a single variable in those clinical studies [42]. The data from two prospective randomized controlled studies on CR TKA revealed that there was significant positive correlation between the postoperative ROM and PTS, with a 2.6° increase in the knee flexion angle for 1° increase in PTS [42]. The kinematics of the PS TKA differ from those of the CR TKA; therefore, researchers believe that their findings were limited to the particular type of the implant and are not observed in all the TKA designs [59].

Based on the result in this study, lower quadriceps forces are required as PTS increased to achieve the postoperative maximal flexion of the knee in PS TKA. In addition, the important finding is that the contact stress on patellar button decreases as PTS increases. Increased PTS induced a more posterior position of the femoral component. The increased PTS can be expected to reduce the quadriceps force and the patellar button contact stress required for deep knee bending to some extent. In addition, decreased quadriceps force and patellar button contact stress showed similar trend with previous study as PTS increased during flexion [20]. However, the contact stress on post in PE insert increased as PTS increased. Anterior impingement between the tibial post and the femoral component was observed at near-full extension with 10° or more of PTS [20]. In other words, excessive PTS can lead to anterior impingement between the tibial post and the femoral component because of the post-cam mechanism, and excessive PTS also leads to increased contact stress on post in PE insert. Our results suggested that the biomechanical effects in PS TKA due to changes in PCO were smaller than those for PTS. Increased PTS causes a positive biomechanical effect, but it may lead to a fracture due to the increased contact stress on post in PE insert.

Our study had several limitations. First, there was a virtual and variable model used in this simulation, and the material properties of soft tissues were obtained from relevant cadaveric studies. These are common methods in computational studies [20–26, 29, 31, 33, 35, 36, 41]. Second, the results could not substitute clinical results and consider patient satisfaction because they were the outcomes of FE analyses. However, the factors (contact stress and quadriceps force) that we analyzed are the main components for evaluating biomechanical effects in computational biomechanics [20, 21, 25, 33–36]. Third, we performed a simulation only for fixed-bearing PS TKA. Therefore, the results from this experiment cannot be considered as representative of all fixed-bearing PS TKAs. Other types of fixed bearings or mobile bearings may yield different results. Therefore, different prostheses and bearing types should be analyzed. Fourth, only the deep-knee-bend simulation was performed, and simulations related to more demanding activities, such as chair rising–sitting, stair climbing–descending, and gait, are necessary for more reliable investigation in future. Finally, this study used linear model for the PE that provided an overestimation in the local stress on PE under plasticization. However, the purpose of this study was to perform a comparative study using the identical model and approach in all the configurations. Thus it highlights the best and the worst configurations. The purpose of the research was not to provide accurate local values of stress or to determine how much changes in PCO and PTS would be necessary to become clinically relevant but only to provide an indication of what could be the stress increases or decreases due to changes in PCO and PTS and which configuration could yield the lowest stress.

In conclusion, changes in PCO led to positive and negative effects on the contact stress on the PE insert and patellar button and the forces on the quadriceps muscle and patellar tendon. However, changes in the forces on the quadriceps muscle and patellar tendon were smaller than those for PTS. In addition, increase in PTS contributed to an improved exercise efficiency of the quadriceps muscle and reduced contact stress of the patellar button. However, excessive PTS should be avoided to prevent fracture in the tibial post.
